# Design and Prestudy Assessment of a Dashboard for Presenting Self-Collected Health Data of Patients With Diabetes to Clinicians: Iterative Approach and Qualitative Case Study

**DOI:** 10.2196/14002

**Published:** 2019-07-09

**Authors:** Alain Giordanengo, Eirik Årsand, Ashenafi Zebene Woldaregay, Meghan Bradway, Astrid Grottland, Gunnar Hartvigsen, Conceição Granja, Torbjørn Torsvik, Anne Helen Hansen

**Affiliations:** 1 Department of Computer Science UiT The Arctic University of Norway Tromsø Norway; 2 Norwegian Centre for E-health Research Tromsø Norway; 3 Department of Clinical Medicine UiT The Arctic University of Norway Tromsø Norway; 4 Department of Neuroscience Norwegian Electronic Health Record Research Centre, Faculty of Medicine and Health Sciences Norwegian University of Science and Technology Trondheim Norway; 5 Department of Community Medicine UiT The Arctic University of Norway Tromsø Norway; 6 Centre for Quality Improvement and Development University Hospital of North Norway Tromsø Norway

**Keywords:** dashboard, self-collected health data, diabetes, mHealth, decision support system

## Abstract

**Background:**

Introducing self-collected health data from patients with diabetes into consultation can be beneficial for both patients and clinicians. Such an initiative can allow patients to be more proactive in their disease management and clinicians to provide more tailored medical services. Optimally, electronic health record systems (EHRs) should be able to receive self-collected health data in a standard representation of medical data such as Fast Healthcare Interoperability Resources (FHIR), from patients systems like mobile health apps and display the data directly to their users—the clinicians. However, although Norwegian EHRs are working on implementing FHIR, no solution or graphical interface is available today to display self-collected health data.

**Objective:**

The objective of this study was to design and assess a dashboard for displaying relevant self-collected health data from patients with diabetes to clinicians.

**Methods:**

The design relied on an iterative participatory process involving workshops with patients, clinicians, and researchers to define which information should be available and how it should be displayed. The assessment is based on a case study, presenting an instance of the dashboard populated with data collected from one patient with diabetes type 1 (in-house researcher) face-to-face by 14 clinicians. We performed a qualitative analysis based on usability, functionality, and expectation by using responses to questionnaires that were distributed to the 14 clinicians at the end of the workshops and collected before the participants left. The qualitative assessment was guided by the Standards for Reporting Qualitative Research.

**Results:**

We created a dashboard permitting clinicians to assess the reliability of self-collected health data, list all collected data including medical calculations, and highlight medical situations that need to be investigated to improve the situation of the patients. The dashboard uses a combination of tables, graphs, and other visual representations to display the relevant information. Clinicians think that this type of solution will be useful during consultations every day, especially for patients living in remote areas or those who are technologically interested.

**Conclusions:**

Displaying self-collected health data during consultations is not enough for clinicians; the data reliability has to be assured and the relevant information needs to be extracted and displayed along with the data to ease the introduction during a medical encounter. The prestudy assessment showed that the system provides relevant information to meet clinicians’ need and that clinicians were eager to start using it during consultations. The system has been under testing in a medical trial since November 2018, and the first results of its assessment in a real-life situation are expected in the beginning of next year (2020).

## Introduction

Personal health information, such as data generated by sensors or data collected by patients themselves through their diaries, contains important information regarding the people’s daily lifestyle. Previous studies have shown that clinicians can use these patient data to provide tailored medical services, especially for patients with chronic diseases [1-3], and that 60% of the patients are open to providing real-time access to their self-collected health information [4]. The use of self-collected data is especially relevant for patients with diabetes, because they often have to adhere to complex treatment regimes. If, for example, a patient is treated with insulin, the dosage has to be adjusted in concordance with not only the calorie intake, but also other factors such as physical exercise [5] and undercurrent disease [6]. Patients with diabetes and physicians have traditionally relied on analog diaries, but as personal computers and smartphones have become commonplace, there has been an explosive increase in the use of digital diaries and wearables [7,8]. In addition, several research projects and private companies are providing solutions to allow clinicians to consult data collected by the patients themselves [2,9]. However, none of these solutions are widely used, mainly because they are proprietary and require specific hardware and software to collect and access the data. This makes it difficult to provide fluid integration between such devices and the physicians’ existing tools and constitutes an important barrier of acceptance for the introduction of these types of data [10].

This paper is part of the “Full Flow of Health Data Between Patients and Health Care Systems” project, which focuses on integrating self-collected health data into consultations in Norway using diabetes and Fast Healthcare Interoperability Resources (FHIR) as a case.

Major health care actors such as Epic Systems Corporation and Cerner propose application programming interfaces relying on FHIR standards [11,12]. Open source projects such as OpenMRS and Open mHealth also provide access to FHIR resources [13,14], and studies propose to use FHIR to improve the health care sector [15]. Norwegian electronic health records (EHRs) are currently working on implementing FHIR standards in their respective solutions [16], but none of them are ready to manage FHIR resources today, as they are not able to receive and display FHIR data. We therefore provided clinicians with a standalone dashboard (ie, view providing key performance indicators) displaying the patients’ self-collected health data to be used as an addition to their current EHR.

Even if self-collected data could be seamlessly integrated, user acceptance is not guaranteed. Patients with diabetes can collect large amounts of data. If the data cannot be presented in an efficient way, it cannot be efficiently comprehended, severely diminishing its usefulness [17-20]. Many physicians struggle to obtain an overview of constantly expanding EHRs. The introduction of a potentially large amount of new data that the physicians are not used to utilizing must therefore be handled with great care, as even minor ill-considered implementation details can have a huge negative impact [18-20]. Optimal presentation of health data depends on the information needed by the clinicians. There is no optimal way of presenting clinical data, because these needs vary a lot [21-25].

This paper presents the design of a dashboard for displaying the self-collected health data from patients with diabetes and describes how the user interface attempts to meet the clinicians’ information needs. Furthermore, the paper presents the prestudy assessment of the dashboard by clinicians.

## Methods

### Phases

**Figure 1:**
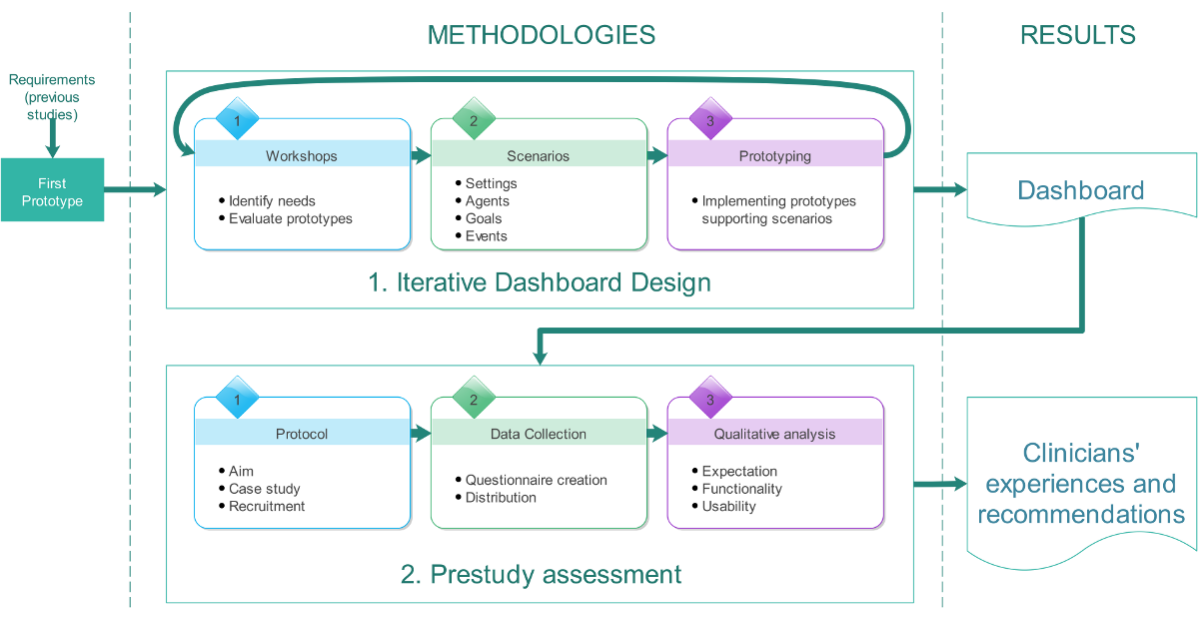
The two main phases of the study, with their components and results.

In the two main phases of the study, we used different methodologies: iterative dashboard design and prestudy assessment (Figure 1). The iterative design phase supported the conception and implementation of the dashboard, while the prestudy assessment was used to collect the clinicians’ experiences with the developed dashboard as well as their recommendations.

Based on previous studies by the authors [26,27], we created the first prototype of the dashboard to be used as a first input for the iterative design process. The information collected from the studies [26,27] was used to identify the data required during diabetes consultations and to define the requirements for the graphical user interface (GUI) of the dashboard.

### Iterative Dashboard Design

The development of the dashboard followed a three-step iterative process to approach the following primary objectives: (1) identify the needs of both patients and clinicians regarding information with clinical relevance during a consultation, information suitable to be collected by patients, and how to present the information in the GUI in order to improve its usability during consultations; (2) evaluate early prototypes and propose adjustments; and (3) develop prototypes based on the proposed adjustments identified in step two.

To achieve these objectives, we organized facilitated workshops, supported by open-ended discussions, to approach specific tasks in rapid development cycles.

### Facilitated Workshops and Open-Ended Discussions

Facilitated workshops are sessions bringing users, stakeholders, and partners together to define and evaluate product requirements [28].

We organized two facilitated workshops using a participatory design approach [29] involving four of the authors (AGi, AGr, EÅ, and AH), four clinicians (two nurses and two doctors who have worked with patients with diabetes), and two patients with diabetes. The clinicians and patients were recruited by our partner—the University Hospital of North Norway. Different methodologies were used during these workshops, namely, brainstorms, idea storms, and go-rounds, to balance creativity and problem-solving tasks and to reduce the pressure on the patients by allowing everyone to speak in turn. The facilitated workshops lasted 3 hours each, and participants were invited to use their own experiences to contribute to the workshops’ primary objectives. The majority group decision–making technique was employed during these sessions.

In addition to the facilitated workshops, we organized a total of 11 sessions with open-ended discussion—three focused on mathematical models to use for medical and statistical calculations and involved two computer scientists; four focused on targeting the GUI usability, namely, the information to be displayed, which was attended by one computer scientist and one GUI expert; two focused on a first assessment regarding the medical relevance of the information displayed, which was joined by a computer scientist and a general practitioner; and two focused on the evaluation of the dashboard prototype against the requirements and involved four of the authors (AGi, AGr, EÅ, and AH).

### Scenarios

We used a simulation-type scenario approach to model real-life situations and narratives [30]. The modelling process relied on a taxonomy containing four elements that were used for each scenario. These elements were as follows:

1. Settings: the context and the situation of the scenario
2. Agents: those who participate in the scenario
3. Goals: the functional targets of the scenario
4. Events: the actions taken by the agents during the scenario

The detailed information concerning the three main scenarios was defined together with the participants during the first facilitated workshop. We chose to use a scenario approach because it facilitates the cooperation of the participants during the facilitated workshops, who can see themselves in the situations and evoke their own experiences, and it simplifies the design process of the dashboard by providing concrete and flexible situations [31].

### Prototyping

The prototyping phase consisted of implementing the dashboard to support the given scenarios by using computer-generated data that express the data requirements for the scenarios.

The dashboard was then built to achieve the objectives described in the scenarios. An agile development process [32] was exclusively used for this task, as evolution, changes, and adaptability were necessary, considering the continuous inputs provided by the workshops. The implementation relied on Java Enterprise Edition 8, Java Server Faces 2.2, and Glassfish 5. The developed prototypes were assessed during the workshops and improved during each iteration of the design process.

Once the authors and participants in the workshops decided that the dashboard was satisfactory to be used in a real situation, we stopped the iterative design process and selected the last prototype for a prestudy assessment by different clinicians.

### Prestudy Assessment of the Dashboard by Clinicians

#### Protocol

The design of the prestudy assessment was guided by the Standards for Reporting Qualitative Research checklist to enhance the organization and reporting of this study [33]. The aim of the prestudy assessment was to evaluate the pertinence of the functionalities presented in the dashboard GUI and its usability prior to a medical trial.

We used a case study approach, organizing a total of five workshops in health care offices (hospital and general practitioner [GP] office), each involving one to four clinicians, accounting to a total of 14 clinicians, and one or two researchers. The 14 clinicians were recruited through our partner, the University Hospital of North Norway, or by direct contact initiated by us; none participated in the dashboard design and all are currently participating in the medical trial. We were limited in the number of participants to include due to external factors (eg, time constraints and unavailability of further participants).

During the workshops, we presented the FullFlow system, which included the last prototype of the dashboard, by using the self-collected health data from one in-house researcher who has type 1 diabetes (an exemption was obtained from the local ethics committee: Ref 2018/719 [34]), hereafter referred to as Research Patient. We extracted these data from the Research Patient’s Diabetes Diary to fill the FullFlow system, using the Diabetes Share Live solution to transmit the data in a way similar to that used in a previous study [27]. The use case presented in the workshops was based on the Research Patient’s real-life diabetes data (ie, insulin intake, carbohydrate intake, blood glucose values, physical activities, weight, medication, and personal aims) and is similar to one scenario created in the dashboard design process. The Research Patient participated in all workshops, where he could explain the different values displayed in the dashboard and answer questions regarding his lifestyle and the recorded values.

#### Data Collection

During the workshops, we distributed a paper-based questionnaire to the participants after presenting the system and letting the clinicians test it. We then collected the questionnaires at the end of the session. The first and second (AGi and EÅ) authors designed a specific questionnaire based on the System Usability Scale [35] and the Computer System Usability Questionnaire [36].

We decided to use a custom questionnaire, as the assessment did not permit inclusion of important usability factors due to a lack of clinical context such as patient-clinician relationships. Given that the questionnaire was administered to the participants before the study, we wanted to provide open-ended questions to obtain important feedback for this iterative process before starting the medical trial. The questionnaire contained four questions about the system and the role of the user (eg, nurse):

- Q1a: Do you think the system will be useful during consultation? Q1b: Potential comments.
- Q2a: Would you like to have more information delivered by the FullFlow system? Q2b: Potential comments.
- Q3a: Would you like to remove or hide information currently delivered by the FullFlow system? Q3b: Potential comments.
- Q4: Do you have any feedback you would like to offer?

#### Qualitative Analysis

The first author (AG) performed a qualitative analysis based on three keywords: *expectation*, *usability*, and *functionality*. In our context, we defined *expectation* as a general belief that positive or negative outcomes could occur in clinical settings by using our proposed system. The use of this term was inspired by the work of Bialosky et al [37]. We used the seven notions provided by Vázquez-García et al [38] to define *usability*: knowability (user can understand, learn, and remember how to use the system), operability (capacity of the system to accommodate users with different needs), efficiency (capacity of the system to produce appropriate results), robustness (capacity of the system to resist error), safety (capacity of the system to avoid risk), and satisfaction (capacity of the system to generate interest in users). We used the definition proposed by Salleh et al [39] to describe *functionality*: a set of functions and their specified properties. We then used the feedback to improve the system before starting the medical trial. We used the feedback obtained in order to improve the system before starting the medical trial.

## Results

### Overview

**Figure 2:**
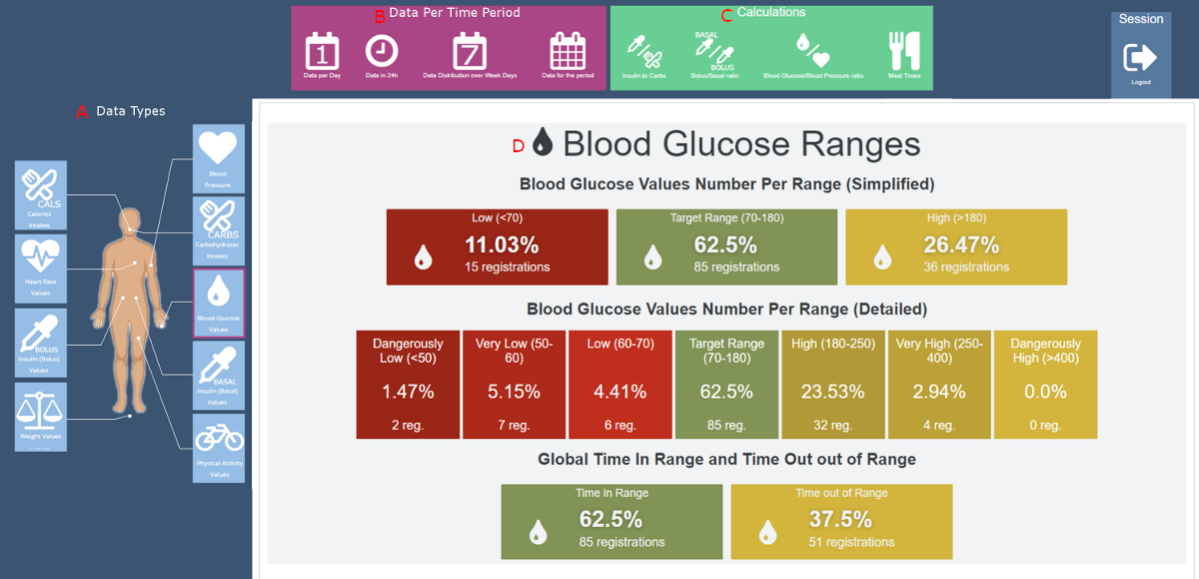
First prototype of the FullFlow dashboard system.

From previous studies, we identified eight relevant data types for diabetes consultation—blood pressure, calories, carbohydrates, heart rate, blood glucose, insulin, weight, and physical activity (Figure 2 A)—and relevant medical calculations such as insulin-to-carbohydrate (I:C) ratio and basal insulin to bolus insulin ratio (Figure 2 C). As a requirement for the GUI, we identified the need to present the data in different time frames (per hour, per day, per week, and for the complete period [Figure 2 B]) and the use of a color scale to illustrate data ranges (Figure 2 D).

### Iterative Dashboard Design

#### Facilitated Workshops and Open-Ended Discussions

The first prototype was presented to the participants in the first facilitated workshop. Based on this prototype and their own experiences, the participants suggested improvements to both data, functionalities, and GUI. The suggested improvements were translated into requirements and implemented in the prototype presented during the second workshop. The improvements suggested during the second workshop were used as requirements during the development of the final prototype. The requirements identified are summarized in Table 1.

#### Scenarios

We created three main scenarios (Table 2); this was considered a manageable number of scenarios for the workshops and open-ended discussions while still allowing diversification of the situations.

**Table 1:** Summary of the requirements defined based on suggestions from the participants in the facilitated workshops and their description.

Requirements	Description
R1: Displaying data collected by patients	At least blood glucose, blood pressure, insulin (bolus/basal), medication, carbohydrates, calories, and physical activity. Being able to accept new data types (eg, menstruation, ketones, and polypharmacy) would be a plus. The system shall inform clinicians if the patients register life goals (eg, what they are focusing on in their daily self-management).
R2: Quantify data collected by patients	The system will notify which data have been collected by the patients and quantify them.
R3: Displaying data collection period	The system will provide clinicians the length of time during which patients collected their data.
R4: Variabilities in the patients’ data values	The system will be able to present a variability value for all data types to indicate how much these values diverge.
R5: Medical calculations	The system will be able to provide medically relevant information (eg, insulin-to-carbohydrate ratio and insulin sensitivity).
R6: Grading data reliability	The system will permit clinicians to know immediately if the data collected by the patients are reliable (ie, worth their time consulting the data).
R7: Hiding eA_1c_^a^	Removing eA_1c_ from the graphical user interface.
R8: Reduce complexity of blood glucose ranges	The system will use the simplified (3 levels) blood glucose range.
R9: Consulting all self-collected health data at once	The system will present all self-collected health data at once in a graph.
R10: Pattern recognition	The system will ease identifying patterns in patients’ lifestyle per day, per week, and for the whole period (eg, hyperglycemic events each day after dinner).
R11: Bridge to existing data	The system shall provide information clinicians can assess by comparing existing data to the self-collected health data.
R12: Overview of the patients’ situations	The system will be able to inform clinicians about what the patients struggle with, what they manage, etc.
R13: Visual helper	The system will provide information about which data are in and out of range.

^a^eA_1c_: estimated hemoglobin A_1c_.

**Table 2:** Scenarios created to support the user requirements. Settings: the context and situation of the scenario. Agents: actors in the scenario. Goals: targets of the scenario. Events: the actions taken by the agents during the scenario.

Taxonomy	Scenario 1	Scenario 2	Scenario 3
Settings	Patient has nightly hypoglycemic events. The patient has an appointment with a diabetes nurse to discuss his situation and therefore collected health data for 1 month prior to the appointment. The patient uses finger pricks and an insulin pen.	Patient struggles with carbohydrate counting and always ends up in hyperglycemia after meals, despite using a hybrid closed-loop system (continuous glucose monitor and a pump). Patient also reaches hypoglycemic levels after the insulin action (“yoyo” effect). Patient has an appointment with a dietitian after having collected 1 week of data.	Patient always has high fasting blood glucose levels, despite being on medication and following cooking courses. Patient has a meeting with his general practitioner after collecting 2 weeks of data.
Agents	Patient with type 1 diabetes and diabetes nurse	Patient with type 1 diabetes and dietitian	Patient with type 2 diabetes and general practitioner
Goals	The system should show the hypoglycemic events and identify the nightly trends. The system should show the insulin dosages and the carbohydrate intakes to help the nurse identify possible points of action.	The system should show the relationship between meal intakes, insulin-on-board levels, and blood glucose levels.	The system should show the high glucose situations, the calorie intakes that are above the recommended levels, the patient’s lack of physical activity, the high blood pressure, and that the patient sometimes forgets to take his medication.
Events	Patient registers, on an average, per day: 10 blood glucose values, 4 carbohydrate intakes, 6 insulin injections (2 basal, 4 bolus), and 10 minutes of physical activity. Nurse discusses the patient’s hypoglycemic events with him and consults the data using the FullFlow dashboard.	Patient registers, on an average, per day: 288 blood glucose values, hourly insulin bolus dosage, and 5 carbohydrate intakes. Dietitian discusses with the patient his “yoyo effect” after meals and consults the self-collected health data using the FullFlow dashboard.	Patient registers, on an average, per day: 1 blood glucose value, 2 medication intakes, and 5 calorie intakes. Patient also has: 2 weight registrations, 1 blood pressure registration, and 3 physical activity registrations (<10 minutes). General practitioner discusses the situation with the patient and uses the FullFlow system to get an overview of his situation.

#### Final Prototype

**Figure 3:**
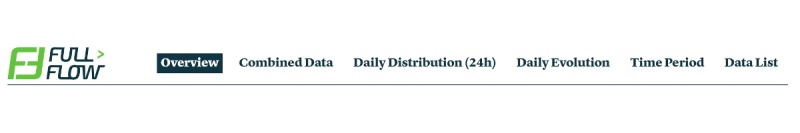
Dashboard menu.

We provide an example of the dashboard based on the self-collected health data obtained from the Research Patient, which was similar to the use case presented to clinicians in the preassessment study. The proposed dashboard contains six main sections accessible from a menu displayed at the top of the page (Figure 3):

1. The *Overview* contains information regarding the data reliability, the data collected, the patients’ personal goals, and a list of noticeable events and their potential causes. It is the landing page of a FullFlow report.
2. The *Combined Data* displays all the quantifiable data sent by the patients in combination with the calculated information for the whole period in a unique graph.
3. The *Daily Distribution* distributes all quantifiable data per hour in multiple graphs (one graph per data type).
4. The *Daily Evolution* summarizes the data per day in multiple graphs (one graph per data type).
5. The *Time Period* displays the data for the whole period in multiple graphs (one graph per data type).
6. The *Data List* lists all the data collected by the patients in a table.

##### Overview Section

**Figure 4:**
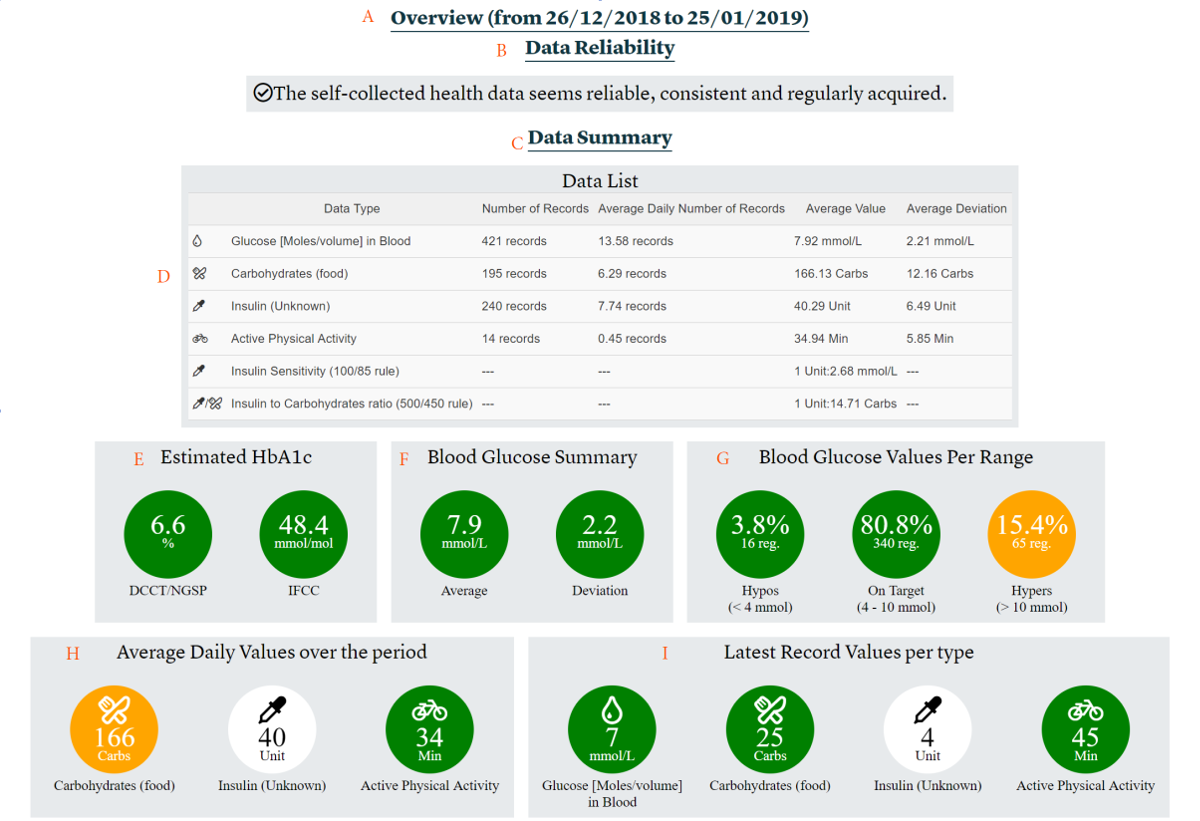
Overview section, part 1. (A) Title and period of time. (B) Data reliability. (C) Summary of the data. (D) List of all the data collected by the patients. (E) Estimated hemoglobin A1c. (F) Blood glucose summary. (G) Time in range and time out of range for blood glucose registrations. (H) Average daily values of data collected by the patients for the period. (I) Latest values for each type of data collected by patients.

**Figure 5:**
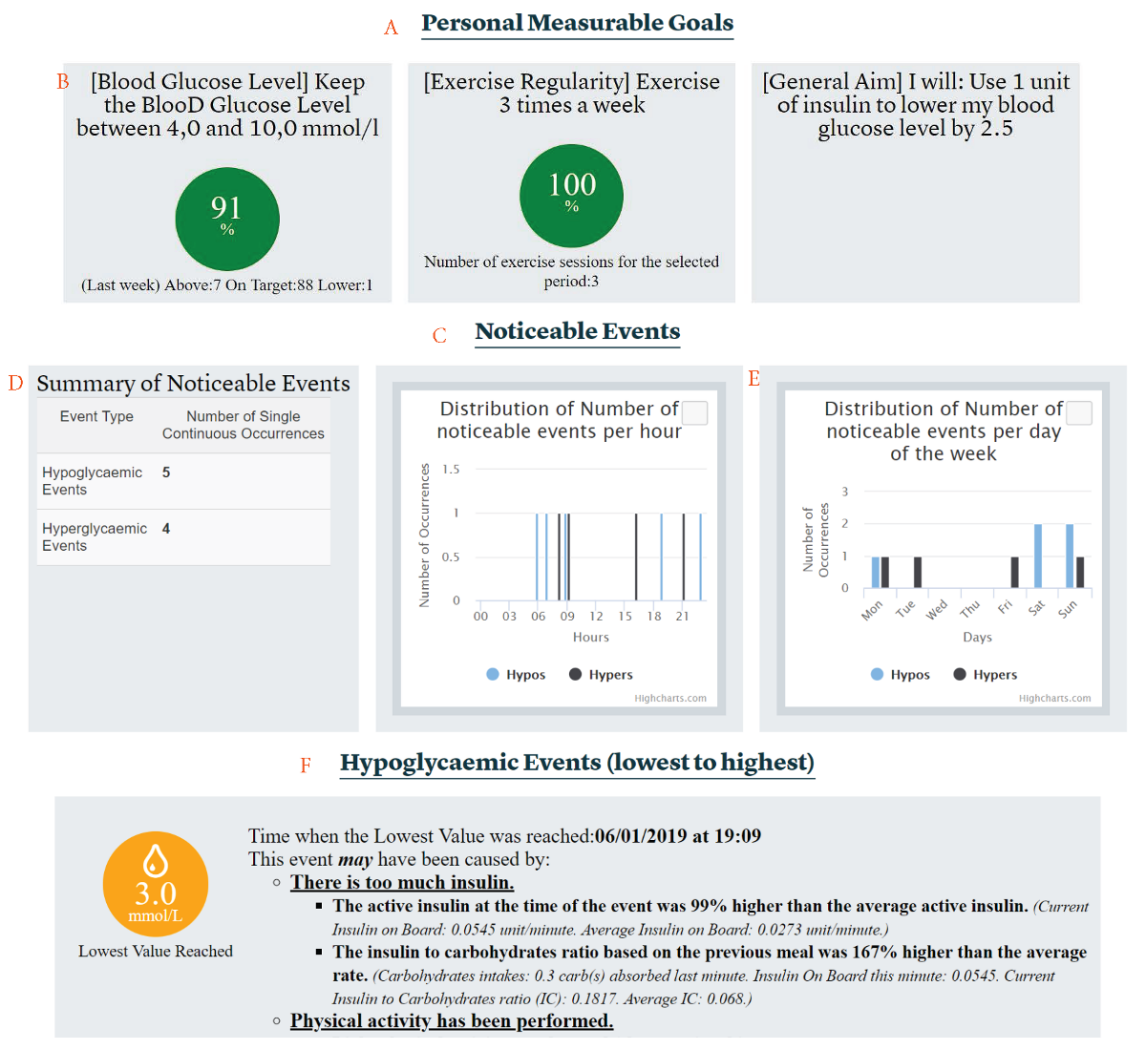
Overview section, part 2. A: List of personal goals defined in the patients’ diary. B: Example of a personal goal. C: List of noticeable events based on the collected data. D: List of events detected organized by type. E. Distribution of event types per day and per hour. F: Example of a noticeable event.

The Overview section provides a summary of all data collected by the patients and the results of the FullFlow analyses (Figures 4 and 5). The objective of this section is to provide an overview of the patients’ situation and the medically related events found to be important to discuss or address, without the need to consult the whole data set. The first data displayed are the time period (Figure 4 A), determined by the first and last FHIR artefacts ordered by date. This addresses the requirement R3 (Table 1).

The second dataset displayed is related to the reliability of the patients’ self-collected health data (Figure 4 B). A knowledge-based module (KBM) grades the reliability of the self-collected health data based on the presence or absence of registered data, potential errors in data values, inconsistencies between data sources, the number of data registrations, and the regularity of the registrations made by the patients. This service addresses the requirement R6 (Table 1) by providing clinicians information about the quality and reliability of data at an early stage of consultation. In this example, the system graded the data as reliable. The system provides a list of issues if the data are graded as not reliable. We explained and illustrated this system in a previous article [40].

The next subsection, the Data Summary (Figure 4 C), first contains a table (Figure 4 D) listing all the patients’ self-collected health data with important calculations for diabetes patients, such as insulin sensitivity and insulin to carbohydrates ratio (I:C), if the data collected permit the calculation of these components. These values are displayed side by side with the ratios submitted by the patients, if available, permitting a simple comparison. The table contains the number of registrations and the average daily number of registrations per day for all types of data collected. The table also provides the average of all the values as well as the pooled SD per data type (called “average deviation” for the clinicians, see Discussion). The pooled SD is calculated using the formula:

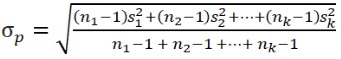



...where *n*_k_ represents the number of registrations for a day and 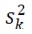
represents the variance for a day. We used the same approach for appropriate data types (eg, not used for blood pressure where the system considers only the latest registered value per day). The table also contains specific diabetes rules, such as the 100/85 rule for estimating the insulin sensitivity (also called “correction factor”) [41] or the 400 rule for estimating the insulin-to-carbohydrate ratio [42]. Patients can also provide this information, and in this case, both collected and calculated values will be listed one above the other for easy comparison. This table addresses requirements R2, R4, and R5 (Table 1).

The next dataset provided is the estimated hemoglobin A_1c_ (eA_1c_) value (Figure 4 E), calculated from the average blood glucose value of all blood glucose registrations, based on the formula proposed by Nathan et al [43]: *eAG*_mmol/L_=1.59* *A*_1c_–2.59, where eAG is the estimated average glucose level in mmol/L and A_1c_ the hemoglobin A_1c_ value. The system calculates the eA_1c_ only if there are at least 3 blood glucose registrations a day and 21 blood glucose registrations in total. This system provides two standards for the eA_1c_ value—National Glycohemoglobin Standardization Program (NGSP; %) and International Federation of Clinical Chemistry and Laboratory Medicine (IFCC) (mmol/mol)—considering that Norway replaced NGSP with IFCC in 2018 [44]. To convert NGSP to the IFCC value [44], we use the following formula:

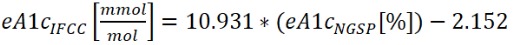



This service addresses the requirement R11 (Table 1) by providing a possible comparison between self-collected health data and laboratory results. However, it also conflicts with the requirement R7 (Table 1). Therefore, we decided to hide these values during the medical trial.

The blood glucose summary (Figure 4 F) displays the average blood glucose value and the pooled SD (same values as in Table 1). The blood glucose values per range (Figure 4 G) display the number of registrations and their percentages per range (low, on target, or high), which are defined as per the standards [45,46]. This addresses requirement R8 (Table 1).

The average daily values (Figure 4 H) display the average of all collected data when appropriate (same values as in Table 1). The final dataset displayed is the last value for each data type the patients have registered (Figure 4 I).

FullFlow grades each piece of information presented in Figure 4 E-I and provides four background color states: green, orange, red, and white. These colors have different meanings: green indicates that the value is in the recommended range, orange indicates that the value is slightly above or under the recommended range, red indicates that the value is out of range, and white indicates that a value is not graded because of a lack of standards or that the value depends heavily on context. The visual representation is inspired by the work of Sim et al [47], who are using a similar grading system, and the work of Diagliati et al [48], who used traffic lights. This grading addresses requirements R13 and R12 (Table 1).

Next, the overview section contains personal goals (Figure 5 A) defined by the patients with or without clinician involvement. Personal goals can be measurable (eg, keeping your blood glucose level between 4 and 10 mmol/L [Figure 5 B]) or nonmeasurable (eg, more proactive). FullFlow provides progress and description for measurable goals. Displaying personal goals addresses requirement R1 (Table 1).

The next section provides information about noticeable events (Figure 5 C). Noticeable events are important events that clinicians and patients should address to improve the health situation of the patients. FullFlow identifies them using KBM in combination with the patients’ self-collected health data and statistical calculations. FullFlow first summarizes the noticeable events by displaying the number of occurrences (Figure 5 D) and distributing the events during the day and the day of the week based on the time, to potentially identify trends (Figure 5 E). Subsequently, FullFlow displays one event at a time, ordered from the most to the least serious, and provides potential causes and explanations for them (Figure 5 F). This section includes other medical conditions related to blood pressure or sleeping pattern in addition to hypoglycemic and hyperglycemic events shown in Figure 5. We described the KBM in detail in a previous article [40]. Noticeable events address requirement R12 (Table 1).

##### Combined Data Section

**Figure 6:**
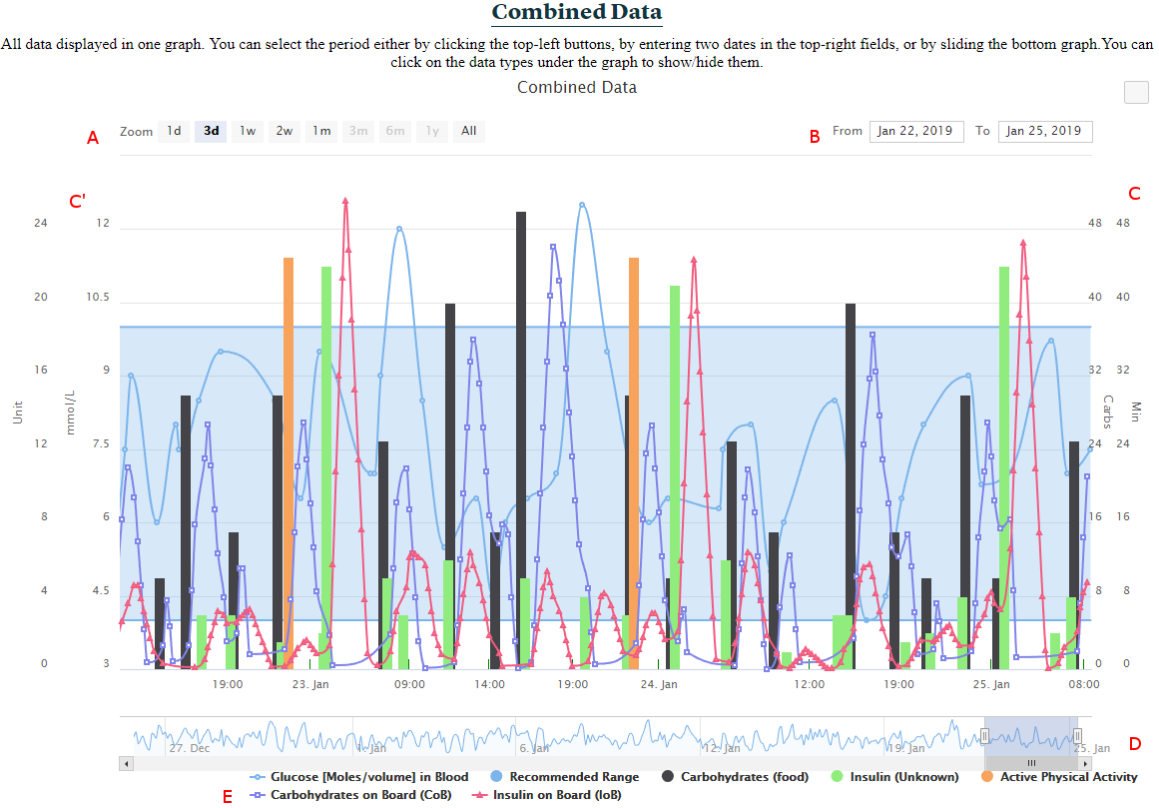
Combined data. (A) Period selection by predefined time length. (B) Period selection by dates. (C, C'): Multiple y-axes. (D) Period selection by range selector. (E) List of all data types represented in the graph.

The combined data section presents all the quantifiable data available in FullFlow (self-collected health data and calculations), as shown in Figure 6. This graph is based on the Highstock library [49] and addresses requirement R9 (Table 1).

Clinicians can change the timeframe by selecting a start and an end date (Figure 6 B) and selecting a predefined time length such as 3 days or 1 week (Figure 6 A) or by sliding, extending, or narrowing the data range selector (Figure 6 D). Clicking on a data type in the lowest part of the graph hides or shows the data type in the center of the graph, allowing clinicians to focus on what they would like to analyze (Figure 6 E). The vertical axes are built automatically (either left or right, Figure 6 C and 6C’) depending on the data type available. The frequency of measurements or the data type extracted from Logical Observation Identifiers Names and Codes or the Systematized Nomenclature of Medicine Clinical Terms contained in FHIR artefacts define the data representation in the graph. Series represent data types having at least 20 registrations per day or being of a specific type, such as blood glucose, while bars represent the rest. Areas represent the reference range of the FHIR artefacts (eg, in-range for blood glucose values) linked to a data type. A mouse hovering above a point shows the exact time and value for all data types with the exact same time. We used the OpenAPS approach to calculate the insulin on board (IoB) [50] and the work of Dana Lewis [51] to calculate the carbohydrates on board (CoB).

##### Daily Distribution Section

**Figure 7:**
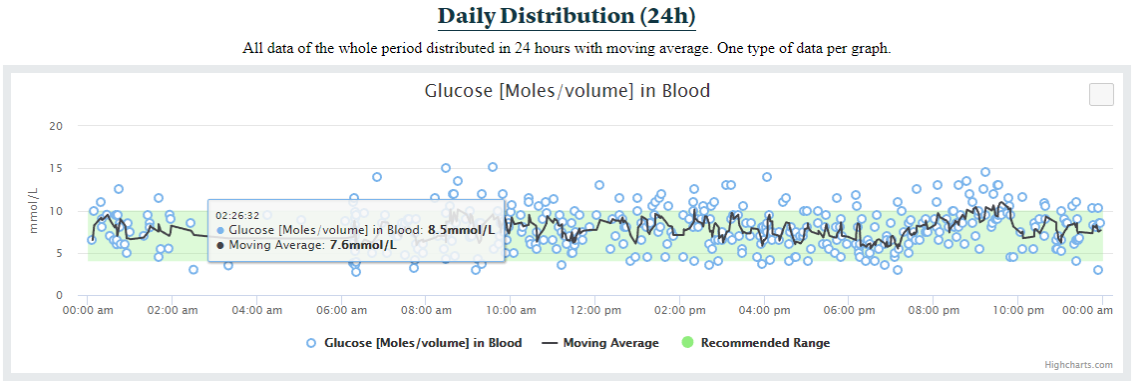
Daily distribution of blood glucose values.

The daily distribution section distributes all the available data in a single day to help clinicians identify daily patterns (requirement R10 in Table 1), such as hypoglycemic events during the nights or hyperglycemic events during the afternoon. This section proposes one graph per data type (Figure 7), which displays all the blood glucose measurements available in a single day. In this example, only finger prick registrations are shown. In addition to displaying the data, FullFlow calculates a moving average of all the values. FullFlow uses either a simple moving average or a weighted moving average, depending on the data type and how patients have collected them (see Discussion for more details). This type of graph also contains reference ranges when provided.

##### Daily Evolution Section

**Figure 8:**
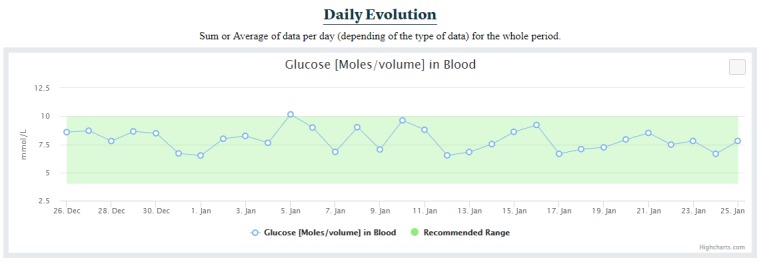
Daily evolution of the blood glucose for the whole period.

The daily evolution section simply presents the sum, the average, or the latest data per day for the whole period, depending on the data type (Figure 8). For instance, blood glucose values are averaged per day, insulin amount values are summed per day, and the latest of the blood pressure values of the day are used for each day. This type of graph also contains reference ranges when provided. Each data type has its own graph.

##### Time Period Section

**Figure 9:**
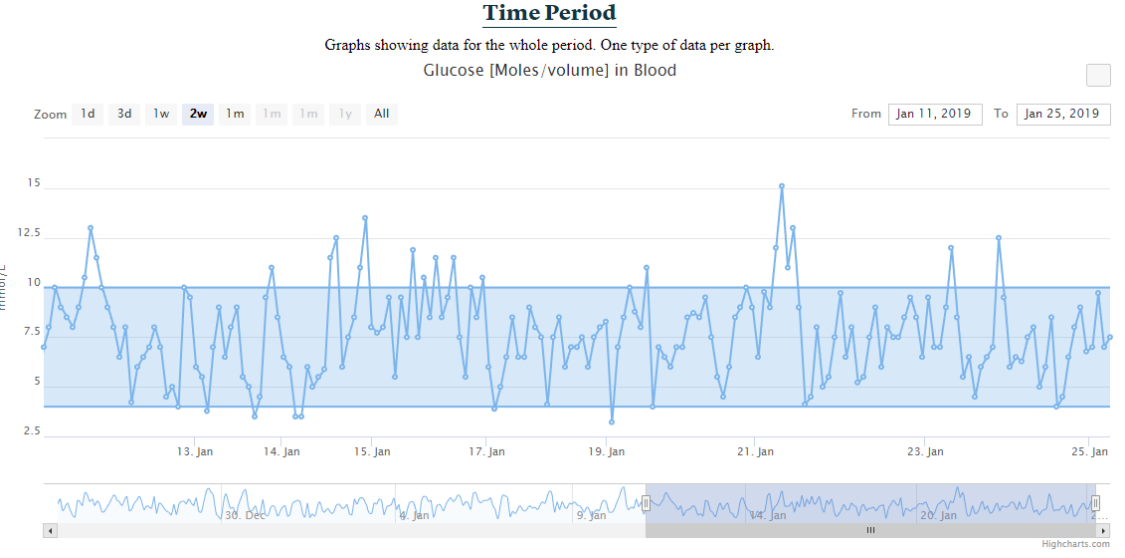
Time period for blood glucose values.

The time period section shows all data available for the whole period by using the same approach as the combined data, except that one graph contains only one data type (Figure 9).

##### Data List Section

**Figure 10:**
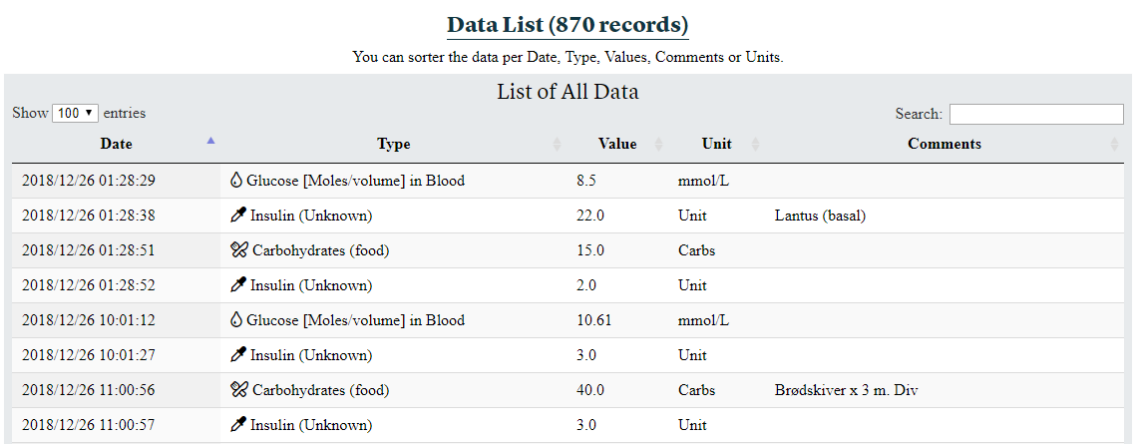
Data list section.

The data list section presents extracted information from all health data self-collected by patients in a list, without the calculated values of the FullFlow, as shown in Figure 10. The section displays the number of registrations made by the patients and shows the date, data type, value, unit, and comment for each entry. Clinicians can order the table by clicking on the head of a column (eg, ordering data per data type) or look up specific registrations using the search field (top right in Figure 10).

The different sections in this dashboard permit the display of any type of data collected by the patients and addresses the requirement R1 (Table 1).

### Prestudy Assessment of the Dashboard by Clinicians

This section presents the assessment of the full system (a combination of the Diabetes Diary [52], Diabetes Share Live [27], and FullFlow) by clinicians, following the approach described in the Methods section. As mentioned in the previous section, the graphical interface was presented without the eA_1c_ value displayed in Figure 4. Multimedia Appendix 1 contains the transcribed answers to the collected questionnaires. The following subsections present the results of the analyses of the questionnaires organized using the taxonomy defined in the Methods section (Data Collection subsection) and concerning the FullFlow system only (the Diabetes Diary and Diabetes Share Live are outside the scope of this study).

#### Participants

Fourteen clinicians participated in the prestudy assessment: nine (64.3%) were GPs, four (28.6%) were diabetes nurses, and one (7.1%) was a dietitian.

#### Pertinence of the Functionalities Provided by the FullFlow System

**Figure 11:**
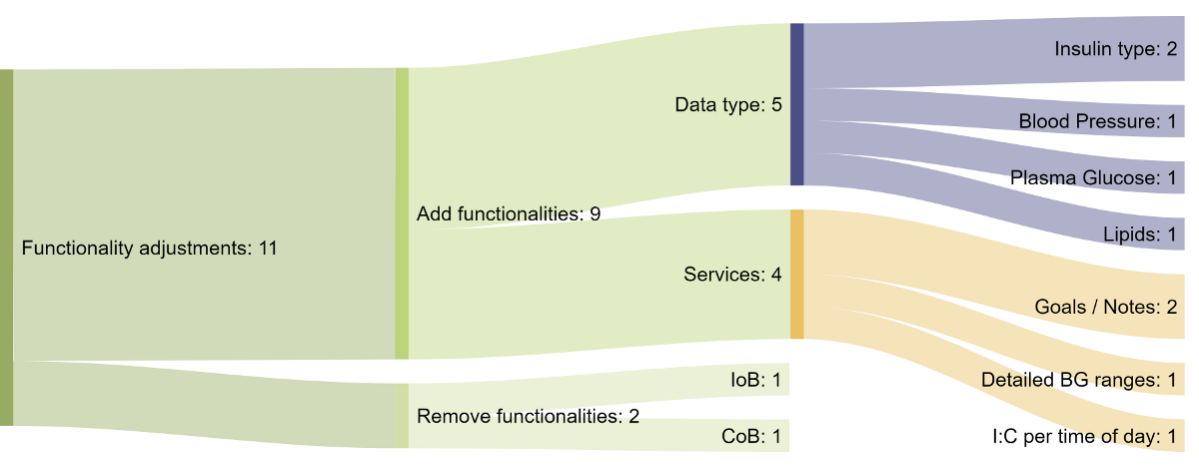
Sankey diagram of the functionality adjustments proposed by the clinicians. Each color corresponds to a specific type of adjustment. Orange: new service; lilac: new data type; light green: remove functionality; green: add functionality; dark green: proposed functionality adjustment. The numbers represent the number of times an adjustment was mentioned. BG: blood glucose; IoB: insulin on board; CoB: carbohydrates on board; I:C: insulin to carbohydrate ratio.

Regarding the relevance of the functionalities provided by the system, the overwhelming majority of the participants (9/14, 64.3%) considered them relevant and would like to keep the system in the current state, without adding or removing any functionalities, as shown in Table 3. Five (35.7%) participants would have liked to add or remove one or more functionalities in the system. Although the majority of the primary health care personnel (GPs) were satisfied with the information available in the system (7/9 or 77.8% would like to keep the system in its current state, while 2/9 or 22.2% would like to alter it), the situation was less clear for the secondary health care personnel (nurses and dietitian), with three (of 5, 60%) clinicians wanting to adjust functionalities and the other two (40%) not wanting to change the system. Regarding functionality alterations, five clinicians proposed 11 points to improve the system and offer more pertinent data (Figure 11).

**Table 3:** Clinicians’ evaluations of potential required adjustments to FullFlow, categorized by the results of the evaluation (to keep or adjust functionalities) and by clinical role (general practitioner, diabetes nurse, and dietician).

Role	General practitioner, n	Diabetes nurse, n	Dietitian, n	Total, n (%)
Adjust functionalities	2	2	1	5 (36)
Keep functionalities	7	2	0	9 (64)

Of the eleven functionality adjustments proposed, nine (of 11, 81.8%) were related to adding new functionalities and the other two (18.2%) were related to removing functionalities. Proposals for adding new functionalities were divided into two subgroups: new services (n=4) and new data types (n=5). New data types would require adding data types not available in the system when they were presented to the clinicians, while adding new services would mean creation of new functionalities using the data currently available in the system. Of the suggested new data types, insulin type (eg, slow or fast acting) was mentioned twice by clinicians, with the suggestion that it be available in both the overview section and the graphs. The other data types suggested were blood pressure, plasma glucose, and lipids. Of the suggested new services, clinicians twice expressed the desire to enter goals and notes directly into the Diabetes Diary of the patients through the FullFlow system. Another clinician requested more detailed blood glucose ranges such as high hypoglycemia in the overview section, and a second suggested displaying I:C values by time of the day (eg, fasting, morning, afternoon, and night). Depending on the situations of the patients, these new data types and services could “help provide more tailored advice” and “facilitate cooperation with the patients,” according to the clinicians. Of the functionalities suggested for removal, one clinician proposed removing IoB and CoB from the graphical interface, suggesting that “they will not have time to investigate this data.”

**Figure 12:**
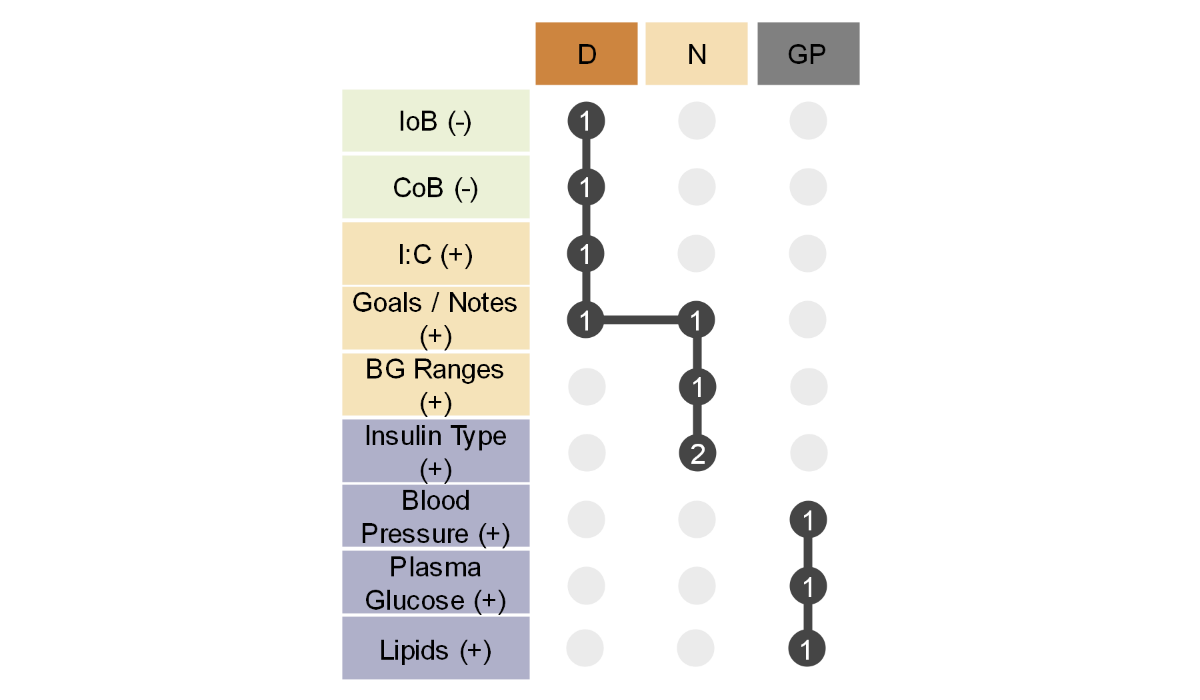
Matrix presenting the correlation between the suggested adjustments and clinical roles (general practitioner, diabetes nurse, and dietitian). The columns represent the clinical roles and use the same color coding as the previous figures (D/orange: dietitian; N/beige: diabetes nurse; GP/grey: general practitioner). The rows represent the adjustments proposed by the clinicians and follow the same categorization and color coding as the previous figure (light green: remove functionality; beige: new service; lilac: new data type). (-) denotes a proposed functionality removal, while (+) denotes a proposed functionality introduction. The dark grey circles represent a suggested adjustment by a specific clinical role and the number of times it was mentioned by that role. The vertical lines represent logical sets, while the horizontal lines denote the intersections of the logical sets, like a Euler diagram. BG: blood glucose; IoB: insulin on board; CoB: carbohydrates on board; I:C: insulin-to-carbohydrate ratio.

Figure 12 shows the correlation between the suggested adjustments and clinical roles. The data show that adjustment needs were disjointed between the primary and secondary health care personnel: The former group expressed the need to add blood pressure, plasma glucose, and lipids to the functionalities of the FullFlow system (mentioned once each), while the latter group did not need them. The secondary health care personnel group proposed adjusting the services available in FullFlow, while the GPs focused only on new data types.

The needs of the dietitian and diabetes nurses intersected, with the proposal of writing goals and notes directly in the Diabetes Diary of the patients via the FullFlow system (mentioned once per group). The nurses proposed recording the insulin type (mentioned twice) and a more detailed blood glucose range (mentioned once) in the FullFlow system. The dietitian was the only clinician to suggest removing features from the FullFlow system (IoB and CoB) and displaying the I:C values by time of the day.

#### Usability of the FullFlow System

One clinician pointed out the possibility of the system being time consuming during consultation, which could reduce its efficiency. Querying the robustness of the FullFlow system, one participant noted that insulin and carbohydrate intake times should be matched in the Combined Data graph. A bug resulting in movement of registrations on the time axis (x) when hiding or showing data types (Figure 6 E) was corrected, and registrations having the same time were shown close to each other.

#### Expectations and Summary

All the participants (14/14, 100%) expected that the presented system—a combination of the Diabetes Diary, Diabetes Share Live and FullFlow—would be useful during their daily consultations. They forecast that the system would be good for all patients, but particularly effective if patients enter enough data regularly in their diaries.

They predicted that three types of patients would be interested in this solution: (1) patients who are interested in technology and self-management; (2) patients concerned about their diabetes and quality of life; and (3) patients living in remote areas, where the usage of the system could support remote consultations and avoid patients travelling several hours for a single face-to-face consultation. One participant mentioned that several patients already use self-management apps, which would ease the introduction of this system.

Overall, the system was very well received by the participants and they were eager to start using it during consultations. However, the participants mentioned that experience using the system will be needed to validate their expectations and clarify the system’s usability and functionality.

## Discussion

### Principal Results

This paper presented a dashboard for displaying patients’ self-collected health data during consultations, using diabetes as a case example. The graphical interface was implemented using continuous feedback from clinicians and patients to minimize possible future user resistance by providing relevant information to meet clinicians’ needs. We limited the potential increase in time consumption due to the usage of this solution by proposing information related to the quality of self-collected health data (identifying whether the data are worth consulting), displaying an overview of the situation of a patient, and identifying important medical events without the need to consult the complete data set.

The prestudy assessment showed that the solution could be effective during consultations, especially if patients live in remote areas or are interested in either mobile technologies or improving their life conditions. The majority of clinicians were satisfied with the current state of the graphical interface, and all clinicians were eager to start using it.

The prestudy assessment also showed that the needs of primary and secondary health care personnel are disjointed: GPs do not need the same data and services as diabetes nurses or dietitians. However, due to the limitations of the Diabetes Diary (see below), their wishes cannot be fulfilled.

### Dashboard Functionalities and Graphical User Interface

The information provided by the KBM module, namely, the grading of the self-collected health data (Figure 4 B), the identification of trends (Figure 5 C-E), and the identification of potential causes of medical events (Figure 5 F) address two of the main barriers of acceptance of introducing self-collected health data into consultation, namely, the distrust of this source of data [53-56] and a time increase in consultation.

The calculations presented in the overview table (Figure 4 D) can facilitate diabetes management [57-60] for both patients and clinicians. We chose to use a table for representing this information, considering that clinicians are accustomed to using tables for visually representing data, which can surpass graphs in certain conditions [61]. We used a standard pooled deviation for illustrating the variability of data type, considering that diabetes, as a chronic disease, is a day-to-day management disease and that a routine (ie, less variability of medical values) can improve the condition of patients drastically [62,63]. For instance, a low glucose variability is more important for diabetes patients than having an in-range hemoglobin A_1c_ for preventing complications [64]. Therefore, providing an indication about how much patients are able to stabilize their blood glucose values during each day is important for them. Although previous studies proposed several methods for measuring glucose variability using SD, coefficient of variability, mean amplitude of glycemic excursion, or continuous overall net glycemic action with CGM, there is a lack of consensus on which method should be used [64,65]. Moreover, these methods have drawbacks when using self-monitoring blood glucose values due to a lack of sufficient and regular number of measurements. Since our system uses available data either from CGM, self-monitoring of blood glucose, or a combination of the two, we are looking for a generic model that can work for all types of available data from the patient. It is quite optimistic to assume that patients self-register data regularly every day, because it reminds them that they are sick [66]; we used pooled SDs to weight the average of each day’s SD. This weighting gives larger groups (days with more registrations) a proportionally greater effect on the overall estimate of the variability [67] and allows us to increase the robustness of statistical calculations. Clinicians agreed to use this approach. Another point to discuss is our decision to use the more accessible term “average deviation” instead of “pooled SD.” We believe that this term will prevent patients and clinicians from being exposed to mathematical concepts in order to understand the value. However, the complete definition, with the formula and explanations of the term, is presented to users if they hover the mouse over the “average deviation” term. Moreover, we expect feedback on this taxonomy from the medical trial.

We decided to use the eA_1c_ functionality, although its use is contested by some authors [68,69] for allowing clinicians to compare the eA_1c_ with the hemoglobin A_1c_ results of the laboratory tests, since previous studies showed that there is a correlation between the hemoglobin A_1c_ and eA_1c_ values [70]. An important deviation between these two values could indicate a poor quality and reliability of the self-collected health data due to, for example, an insufficient number of registrations per day and can therefore be used as one of the indicators of the quality and reliability of the self-collected health data. Today, due to technical restrictions, the FullFlow system cannot integrate EHRs’ data and display the hemoglobin A_1c_ value side by side with the eA_1c_. Clinicians can consult the hemoglobin A_1c_ values in their EHRs and use FullFlow for consulting the eA_1c_. In addition, this approach is used by the American Diabetes Association [71] and MySugr [72] and is cited in the NGSP’s website [73]. However, we decided to hide the eA_1c_ value, considering that clinical workers were concerned that this value can confuse patients in Norway. Nonetheless, the system will still collect the value, allowing us to compare the calculated values against the laboratory test results or the hemoglobin A_1c_ values reported through questionnaires, to determine how this approach fits real situations. The dashboard containing the eA_1c_ may be of interest to clinicians, patients, researchers, and computer scientists.

Regarding the grading of each piece of information (Figure 4 E-I), the system uses different approaches depending on the type of data. For instance, the FullFlow relies on medical standards given by the Norwegian Directorate for Health [74] and international public entities [75] (eg, hemoglobin A_1c_ values) or values we defined during our workshops (eg, grades for the blood glucose in-range values). Some values are not graded, such as the daily amount of insulin used, because each patient follows tailored insulin therapy, depending on physiological conditions such as weight as well as lifestyle factors such as meal times and physical activity [76].

Displaying the patients’ personal goals in the overview section (Figure 5 A) before the noticeable events will help the patients steer the medical consultation toward what they would like to discuss with their clinicians, as some of them are too shy to interrupt the clinicians directly, according to the feedbacks collected in the workshops.

The moving average and weighted moving average used by the daily distribution section (Figure 7) further facilitate the visual detection of patterns by clinicians, which can be useful for improving patients’ lifestyle [77,78]. We are aware of other types of moving averages such as the exponential weight moving average [79] or the Hull moving average for reducing lag [80]. However, we decided to use a simple weighted moving average in the first version of the FullFlow. The decision regarding the usage of a weighted or simple moving average relies on the analysis of the FHIR artefacts. For instance, a blood glucose value obtained from a finger prick has twice the weight of a blood glucose value measured with a CGM, considering that finger pricks are more accurate than the CGMs, which require calibration [81]. The window size for calculating the moving average is set to five registrations to suppress the sheer power the CGM readings have over the self-monitoring blood glucose measurements (ie, five registrations maximum are used for calculating one value of the weighted moving average). This fact remains true even though the CGMs are becoming more accurate [82] and some do not require calibration at all [83].

### Comparison with Previous Studies

The dashboard we proposed differs from others such as MySugr [84], the dashboard of Diagliati et al [48], Carelink by Medtronic [85], the clinical decision system by Sim et al [47], the system proposed by Martinez-Millana et al [86], and the platform proposed by Fico et al [87]. The main differences are listed below:

1. FullFlow does not limit the integration of data to specific companies or types of sensors: finger pricks, CGMs, insulin pens or insulin pumps can all be used by the patients.
2. FullFlow analyzes the data and proposes recommendations regarding potential causes of medical situations.
3. FullFlow provides indicators regarding the reliability of the self-collected health data.
4. FullFlow empowers patients by introducing their personal goals in the medical consultation.

### Limitations

The first limitation is the size of the sample for the design and prestudy assessment phases, in which 18 clinicians and 2 patients participated. Although the sample did not permit involvement of all types of clinical roles to identify their needs and evaluate the graphical interface according to their preferences, it was sufficient for determining that the dashboard is ready to enter a medical trial.

During the prestudy assessment, one of the clinicians mentioned that (s)he was afraid that the system could be time consuming. Although the KBM can, in theory, address this issue, as we explained in a previous article [40], we fear this challenge will greatly impact the medical trial due to the technical solutions chosen.

We know that the chosen patient platform, the Diabetes Diary, is not the optimum app for all diabetes patients, as it lacks important features such as the insulin type, blood pressure, polypharmacy, and integration into glucometers and physical activity trackers for automatic data transmission. These missing features might result in a degradation of the reliability of the data and experience for the patients as well as for the clinicians, who would like to have access to these missing data, as specified in the Prestudy Assessment section. Moreover, the Diabetes Share Live solution platform, which requires many steps to be performed during consultation for viewing the self-collected health data, could degrade the experience of the users. This platform requires eight steps to share the data: (1) patients open the Diabetes Diary, (2) patients wait for the application to give a unique identification code, (3) clinicians open an Internet Navigator, (4) patients give clinicians the unique code, (5) clinicians enter the code on the Webpage, (6) clinicians choose a time period, (7) patients acknowledge the time period given by the clinicians and select the data they want to share, and (8) clinicians consult the FullFlow.

**Figure 13:**
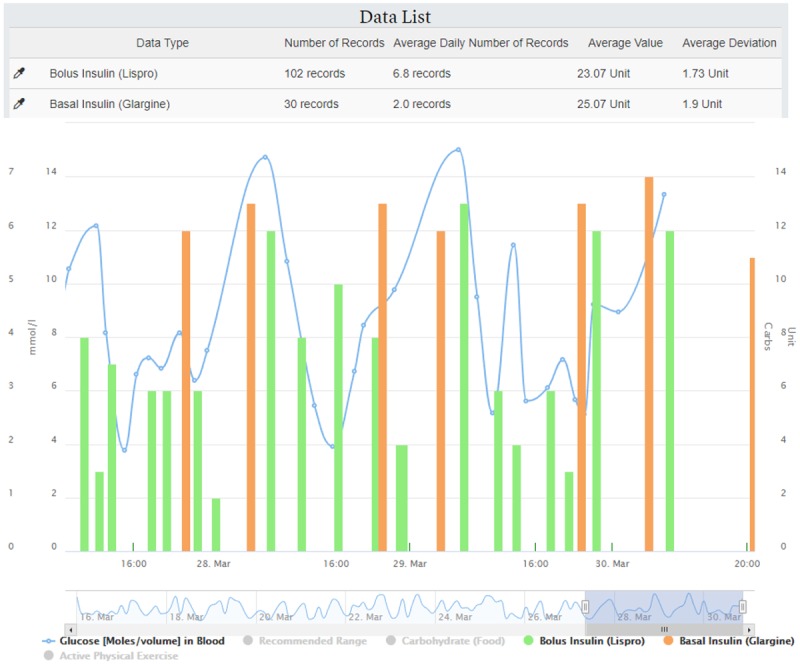
Example of data list and combined data with different types of insulin.

However, the FullFlow system itself is not affected by these limitations and can accept data from several applications and several operating systems. For example, while the insulin type will not be displayed during the medical trial (the system displays “Insulin Unknown”; Figure 4 D and 4 H), the FullFlow differentiates types of insulin and treats them differently when such information is available. Figure 13 shows an example of different insulin types for data collected using the MySugr app, where bolus and basal insulin types are treated as separate entities and combined to calculate the IoB by using different profiles [50]. Multimedia Appendix 2 shows an instance of the dashboard populated with other data types and demonstrated that the system is able to display any FHIR data.

Nevertheless, the medical trial will still allow us to conduct research on the relevance of the information displayed, its potential impact on medical services, and the relevance of the KBM. Although the approach and business rules of the KBM are trusted by the clinicians who were involved in its creation, the medical trial will measure trust in the system during its usage, which will depend on the situation of the patients and the data collected by them. It could also be suitable for remote consultation.

The last limitation concerns the integration of EHR data into FullFlow, which, while planned, is not yet available. Therefore, FullFlow cannot directly show EHR data, such as hemoglobin A_1c_, and clinicians will have to use both systems during consultations. However, while not reaching its maximum potential, FullFlow will still permit the study of the integration of self-collected health data into consultations.

### Future Research

The graphical interface can still be improved in different ways: The table in the Data Summary section could contain information related to the in-range values of each data type and be visually graded like the rest of the overview page (green, orange, red, and white). Shortcuts to the combined graph from a noticeable event could be made, with automatic selection of data to display or hide. It may also be possible to see self-collected health values day by day, with the current day values displayed in a large graph at the top of the page and all other days’ values listed under this graph as smaller graphs, one per day; we could also add daily computational glucose variability using SDs to the top of the overall graph.

We believe that the results from the medical trial, in which clinicians use FullFlow in their daily consultations, are necessary to assess what information is useful to add or remove, before changing the graphical interface. Nevertheless, we believe that the proposed dashboard is a viable temporary solution, and ensuring interoperability of the data using standards and terminologies will allow the independence of the EHRs and permit users to display the information in the ways that benefits most of their users.

The graphical interface could also be improved by adding dual signaling for visually impaired people. For instance, the data summary table in the overview section could integrate visual cues, such as equals signs or arrows pointing up or down, to indicate whether values are in range or out of range. These signs could be added below the values displayed in circles in the overview section or even used as texture.

In addition, reports do not contain information regarding the patients themselves (eg, names or identity numbers). This is due to the usage of the Diabetes Diary and Diabetes Share Live. It will not affect the medical trial, given that clinicians and patients use the system in real time together and clinicians can export the reports to their EHRs, where the patient will already be selected. Notably, clinicians would like to write goals or notes directly into the patients’ apps using the FullFlow system, which is outside the scope of the study at this stage; we would suggest that patients use their mobile apps themselves to directly create the goals defined in collaboration with their clinicians during consultation.

Although the system can read and display any data types as long as they are in an FHIR format, it will use only “registered” data types for advanced services (eg, blood glucose, insulin, blood pressure, and menstruation), such as grading data reliability or exploring potential causes of medical events. The registered data types are listed in another article [40]. We plan to add new business rules for new data types in the future, such as lipids (as requested by a clinician) or foot temperature for early detection of injuries due to diabetic neuropathy. Multimedia Appendix 2 shows an example of the graphical interface containing lipids as “unregistered” data type and six registered data types.

### Conclusions

The designed dashboard could ease the introduction of self-collected health data during medical consultation by providing relevant information about the situation of the patients, the reliability of the data, and important medical events without the need to consult the data in details. Moreover, the designed dashboard could be an effective solution for face-to-face and remote consultations.

A medical trial, started in November 2018, will provide medical context and document user experience and medical outcomes through usage logs, interviews, and surveys and will help us adjust and improve the dashboard in terms of its graphical interface and functionalities. The results are expected in the beginning of 2020.

## Appendix

Multimedia Appendix 1 Transcribed answers to the collected questionnaires.

Multimedia Appendix 2 Example of the graphical interface with different data types.

## Abbreviations

CGM: continuous glucose monitor
CoB: carbohydrates on board
eA _1c_: estimated HbA_1c_
EHR: electronic health record
FHIR: Fast Healthcare Interoperability Resources
GP: general practitioner
GUI: graphical user interface
I:C: insulin-to-carbohydrate ratio
IFCC: International Federation of Clinical Chemistry and Laboratory Medicine
IoB: insulin on board
KBM: knowledge-based module
NGSP: National Glycohemoglobin Standardization Program
