# Supplementary material for: Design and Prestudy Assessment of a Dashboard for Presenting Self-Collected Health Data of Patients With Diabetes to Clinicians: Iterative Approach and Qualitative Case Study
Source: JMIR Diabetes. 2019 Jul 9;4(3):e14002. doi: 10.2196/14002 (PMC6647758; doi:10.2196/14002)
Supplement: Multimedia Appendix 1 [file diabetes_v4i3e14002_app1.pdf]

Role	Q1	Q1b	Q2	Q2b	Q3	Q3b	Q4
Dietitian	Yes	Useful for patients *who have Android phones* and are technologically interested only.	Yes	Possibility to use the system to define and write goals.<br>More detailed insulin to carbohydrates ratio at different times of the day (breakfast, lunch, afternoon, evening)	Yes	Active Insulin<br>Carbohydrates on Board	Match between time for insulin and time for carbohydrates intakes
Diabetes Nurse	Yes	I think the system will be useful, but I have few consultations per day.<br>This is a good option for users who like to use mobile application for registrations.	No	*Patients would like to enter non-diabetes related drugs taken at fixed time, and this could be automatic and/or easy to enter*	No	Not now	*Have patients' opportunity to write the type of carbohydrates they eat (the amount may not be always enough)*<br>This solution may be good for everyone, and especially for patients living at long distances and for patients who follow technology development.
Diabetes Nurse	Yes	*The Diabetes Diary can replace written diary.*<br>Several patients use other apps today and are used to them and their functionalities	No	I think the system can be time consuming. No more information should be added now.	No	-	When hovering over the carbohydrates registration show what the patients have eaten.
Diabetes Nurse	Yes	-	Yes	Insulin type.	No	Need experience to answer this	Notes from the consultation should be possible to add to the diary
GP	Yes	Only for patients who are a bit concerned about their diabetes	No	-	No	-	Must use the system to be evaluated again.
Diabetes Nurse	Yes	It depends if the patient has entered enough data.	Yes	Would like to have the distribution of slow and fast acting insulin.	No	Don't know yet, but I think I'm going to use the Combined data Section the most	What about different colors on the bar for slow-acting and fast acting insulin?<br>On the overview, display blood glucose above 15 mmol/L and under severe hypos (under 2.8 mmol/L) in a separate body.
GP	Yes	-	Yes	Blood Pressure	No	-	Desire to insert lipids
GP	Yes	-	No	-	No	-	-
GP	Yes	-	No	-	No	-	*Have the possibility to register data automatically directly from Strava, blood glucose devices, insulin pen etc.*
GP	Yes	-	Yes	Plasma glucose	No	-	-
GP	Yes	-	No	-	No	-	-
GP	Yes	Can motivate new patients to join in	No	-	No	-	-
GP	Yes	-	No	-	No	-	-
GP	Yes	-	No	-	No	Do not know yet	-

- Q1: Do you think the system will be useful during consultation? Q1b: Potential comments
- Q2: Would you like to have more information delivered by the FullFlow system? Q2b: Potential comments
- Q3: Would you like to remove or hide information delivered by the FullFlow system today? Q3b: Potential comments
- Q4: Do you have any feedback you would like to give to us?

Comments in green concern the Diabetes Diary only and are outside of the scope or the article.
