# Supplementary material for: Design and Prestudy Assessment of a Dashboard for Presenting Self-Collected Health Data of Patients With Diabetes to Clinicians: Iterative Approach and Qualitative Case Study
Source: JMIR Diabetes. 2019 Jul 9;4(3):e14002. doi: 10.2196/14002 (PMC6647758; doi:10.2196/14002)

Example of the graphical interface with different data types. Differences with the screenshots provided within the article:

1) Concerns T2D patient with oral medication, blood pressure and weight;
2) Low data quality;
3) Unregistered data type (Lipids) – manually generated;
4) No Hba1c due to too low number of blood glucose registrations;

Overview (from 05/03/2019 to 12/03/2019)

Data Reliability

ⓘThe quality of the self-collected health data seems uncertain: a significant number of issues are presents.

Hide/Show details

Data Summary

Data List

Data Type	Number of Records	Average Daily Number of Records	Average Value	Pooled Standard Deviation
Lipids measurement	1 records	0.12 records	6.25 lipids	-- lipids
Medication	15 records	1.88 records	1593.75 mg.	-- mg.
Glucose [Moles/volume] in Blood	10 records	1.25 records	7.77 mmol/L	-- mmol/L
Calories	15 records	1.88 records	2475.0 cal	-- cal
Weight	3 records	0.38 records		
Blood pressure	1 records	0.12 records	-- mmHg	-- mmHg
Active Physical Activity	3 records	0.38 records	16.25 Min	-- Min

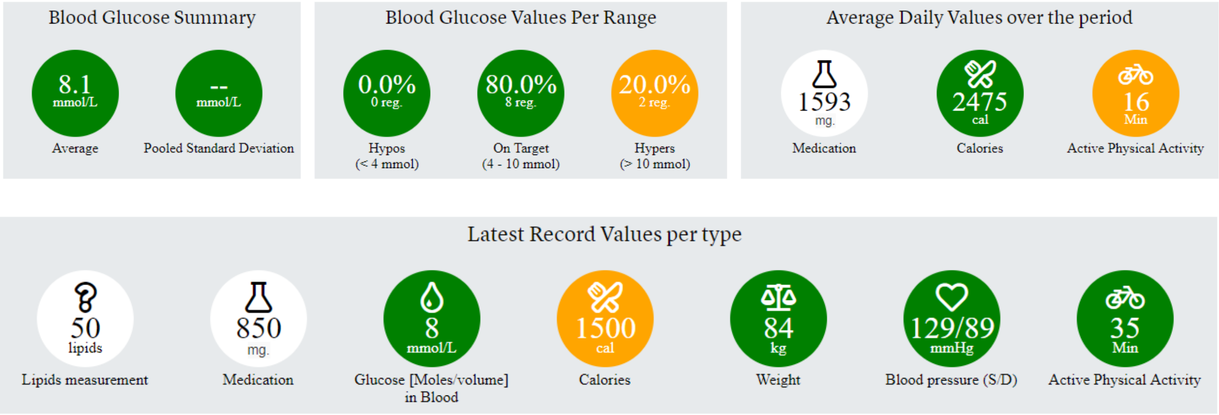

Supplement: Multimedia Appendix 2 [file diabetes_v4i3e14002_app2.pdf]
